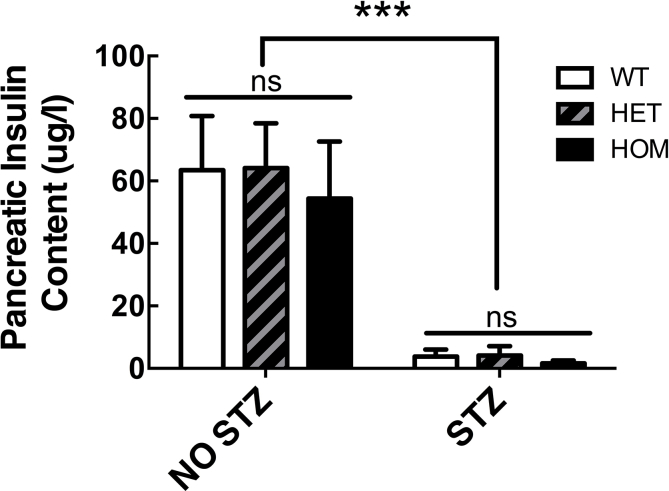# Molecular reductions in glucokinase activity increase counter-regulatory responses to hypoglycemia in mice and humans with diabetes

**DOI:** 10.1016/j.molmet.2018.08.001

**Published:** 2018-08-13

**Authors:** Ali J. Chakera, Paul S. Hurst, Gill Spyer, Emmanuel O. Ogunnowo-Bada, William J. Marsh, Christine H. Riches, Chen-Yu Yueh, S. Pauliina Markkula, Jeffrey W. Dalley, Roger D. Cox, Ian A. Macdonald, Stephanie A. Amiel, Kenneth M. MacLeod, Lora K. Heisler, Andrew T. Hattersley, Mark L. Evans

**Affiliations:** 1 Institute of Clinical and Biomedical Sciences, University of Exeter, United Kingdom; 2 Wellcome Trust/ MRC Institute of Metabolic Science and Department of Medicine, University of Cambridge, United Kingdom; 3 Behavioural and Clinical Neuroscience Institute and Departments of Psychology and Psychiatry, University of Cambridge, United Kingdom; 4 MRC Harwell Institute, Mammalian Genetics Unit, Harwell Oxford, United Kingdom; 5 MRC-ARUK Centre for Musculoskeletal Ageing and NIHR Nottingham Biomedical Research Centre, Nottingham University Hospitals NHS Trust/ University of Nottingham, Nottingham, United Kingdom; 6 Division of Diabetes and Nutritional Sciences, King's College London, United Kingdom; 7 Rowett Institute, University of Aberdeen, United Kingdom

**Keywords:** Glucokinase, Hypoglycemia, Counter-regulation, Glucagon, Epinephrine, Insulin clamp, Maturity onset diabetes of young (MODY), β-cells, ARC, Arcuate nucleus, GE, Glucose excited, GI, Glucose inhibited, GCK, Glucokinase, HOM, Homozygous, HET, Heterozygous, STZ, Streptozotocin, VMN, Ventromedial hypothalamus, WT, Wild type, SF1, steroidogenic factor 1

## Abstract

**Objective:**

Appropriate glucose levels are essential for survival; thus, the detection and correction of low blood glucose is of paramount importance. Hypoglycemia prompts an integrated response involving reduction in insulin release and secretion of key counter-regulatory hormones glucagon and epinephrine that together promote endogenous glucose production to restore normoglycemia. However, specifically how this response is orchestrated remains to be fully clarified. The low affinity hexokinase glucokinase is found in glucose-sensing cells involved in glucose homeostasis including pancreatic β-cells and in certain brain areas. Here, we aimed to examine the role of glucokinase in triggering counter-regulatory hormonal responses to hypoglycemia, hypothesizing that reduced glucokinase activity would lead to increased and/or earlier triggering of responses.

**Methods:**

Hyperinsulinemic glucose clamps were performed to examine counter-regulatory responses to controlled hypoglycemic challenges created in humans with monogenic diabetes resulting from heterozygous glucokinase mutations (GCK-MODY). To examine the relative importance of glucokinase in different sensing areas, we then examined responses to clamped hypoglycemia in mice with molecularly defined disruption of whole body and/or brain glucokinase.

**Results:**

GCK-MODY patients displayed increased and earlier glucagon responses during hypoglycemia compared with a group of glycemia-matched patients with type 2 diabetes. Consistent with this, glucagon responses to hypoglycemia were also increased in I366F mice with mutated glucokinase and in streptozotocin-treated β-cell ablated diabetic I366F mice. Glucagon responses were normal in conditional brain glucokinase-knockout mice, suggesting that glucagon release during hypoglycemia is controlled by glucokinase-mediated glucose sensing outside the brain but not in β-cells. For epinephrine, we found increased responses in GCK-MODY patients, in β-cell ablated diabetic I366F mice and in conditional (nestin lineage) brain glucokinase-knockout mice, supporting a role for brain glucokinase in triggering epinephrine release.

**Conclusions:**

Our data suggest that glucokinase in brain and other non β-cell peripheral hypoglycemia sensors is important in glucose homeostasis, allowing the body to detect and respond to a falling blood glucose.

## HIGHLIGHTS

• Reduced glucokinase (GCK) function increases hormonal responses to hypoglycemia.
• β-cell GCK is responsible for insulin suppression as blood glucose levels fall.
• Brain GCK-mediated hypoglycemia-sensing is involved in epinephrine release.
• GCK-mediated glucagon secretion involves GCK that is not located in brain or β-cells.

## Graphical abstract

**Figure:**
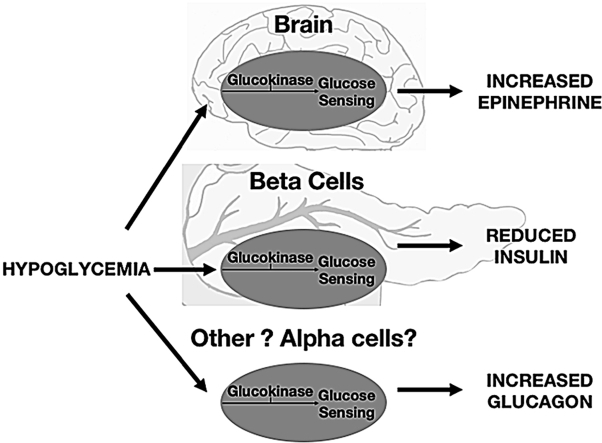

## 1 Introduction

Maintaining blood glucose within an appropriate range is crucial for survival. In diabetes, those who suffer episodes of severe hypoglycemia have an increased risk of death [1], [2]. The brain is especially dependent on an adequate, continuous supply of circulating glucose to fuel metabolism and support function. To defend against falling blood glucose, a series of robust counter-regulatory responses normally prevent hypoglycemia from occurring [3]. These include the ability to switch off endogenous insulin secretion as blood glucose levels start to fall and release the key counter-regulatory hormones glucagon and epinephrine. In order to detect and respond to changes in blood glucose, a network of specialized glucose sensors in the periphery and brain forms an integrated network to trigger counter-regulation as hypoglycemia starts to develop [4].

The glucose-sensing apparatus in pancreatic β-cells includes the low affinity hexokinase glucokinase (GCK), which controls glycolytic flux into downstream metabolic sensing by ATP-gated potassium (K_ATP_) channels. In the canonical β-cell pathway, a fall in glucose is detected as a fall in this metabolic flow, with opening of K_ATP_ channels leading to an early cessation of insulin release from β-cells [5].

If blood glucose falls further, glucagon release during hypoglycemia probably has a redundancy of controlling inputs. α-cells contain GCK and K_ATP_ channels and may sense hypoglycemia directly [6], [7]. α-cells also likely receive information from local paracrine signalling and/or direct contact from β and δ-cells within islets [7], [8], [9], [10]. Additionally, circulating catecholamines and pancreatic neural autonomic innervation may contribute to a coordinated homeostatic glucagon response [11], [12].

Epinephrine release as part of a sympatho-adrenal response to hypoglycemia is driven by increased outflow from the brain to the adrenal medulla [4]. Brain glucose sensors may be glucose-excited (GE) or inhibited (GI) [13]. Counter-regulatory responses may be triggered either by hypoglycemia-induced GI activation and/or GE silencing. Glucose sensing in brain sensors may employ mechanisms similar to those identified in the beta cell including GCK [14], [15], [16], [17], [18], [19], [20].

Here we examined the role of GCK in the hormonal counter-regulatory protection against hypoglycemia, examining responses in mice and humans with defined molecular disruptions (mutations or ablation) of GCK. We hypothesized that reducing GCK activity would reduce glycolytic flux into downstream metabolic sensing pathways and trigger earlier and/or increased counter-regulation compared with controls during controlled matched hypoglycemic challenges. We studied humans and mice, using defined molecular perturbations in the latter to gain further insight into the sites where GCK-mediated glucose sensing plays a role in counter-regulatory responses.

## 2 Material and methods

### 2.1 Human Participants

We studied eight participants with GCK-MODY (Maturity Onset Diabetes of Young due to heterozygous inactivating mutations of the *GCK* gene), comparing responses with data collected from eight healthy controls and six participants with type 2 diabetes matched for fasting plasma glucose with GCK-MODY participants. All three groups were matched for age (±5 years), gender, and BMI (±10%). Data from the group with type 2 diabetes and seven of the healthy control group have been previously published [21]. The subjects in the present report were all studied in random order during the same time period. The characteristics of these participants and basal hormone concentrations are shown in Table 1. Details of identified mutations in GCK-MODY group are listed in Supplementary Table 1. The study was performed in accordance with the Code of Ethics of the World Medical Association (Declaration of Helsinki) and was approved in advance by a local independent ethics committee. All participants gave informed written consent prior to the start of the study.

**Table 1:** Group characteristics and baseline hormone levels for participants undergoing hypoglycemic clamps.

	GCK-MODY	Type 2 diabetes	p-value GCK-MODY vs Type 2 diabetes	Healthy controls	p-value GCK-MODY vs Healthy controls	p-value Type 2 Diabetes vs Healthy controls
Number	8	6	–	8	–	
Female/Male	6/2	4/2	–	6/2	–	
Age	41.5 ± 4.1	46 ± 4.0	0.2	41.5 ± 4.9	0.8	
BMI/kg m^−2^	31.8 ± 2.4	31.7 ± 3.0	0.6	28.8 ± 1.8	0.5	
FPG/mmol L^−1^	7.0 ± 0.6	7.2 ± 1.1	0.9	4.5 ± 0.8	0.0008	0.002
HbA1c/% mmol mol^−1^	6.4 ± 0.5 46 ± 5.5	7.4 ± 1.0 57 ± 11	0.06	5.6 ± 0.5 38 ± 5.5	0.01	
Treatment	Diet 7, Sulphonylurea 1	Diet 2, Sulphonylurea 2, Metformin 2		None		
C-peptide/μmol L^−1^	862 ± 356	1120 ± 566	0.8	694 ± 233	0.2	0.1
Epinephrine/nmolL^−1^	0.29 ± 0.1	0.38 ± 0.2	0.2	0.21 ± 0.1	0.07	0.07
Norepinephrine/nmolL^−1^	2.1 ± 0.2	2.0 ± 0.5	0.4	2.0 ± 0.8	0.9	0.8
Pancreatic polypeptide/pmolL^−1^	15.1 ± 8.1	34.1 ± 43.0	0.4	20.0 ± 16.3	0.5	0.6
Glucagon/pg ml^−1^	96 ± 15	131 ± 45	0.07	98 ± 38	0.8	0.1
Cortisol/nmol L^−1^	295 ± 133	343 ± 209	0.8	255 ± 83	0.6	0.7
Growth Hormone/mu L^−1^	5.1 ± 4.9	1.5 ± 0.9	0.3	2.2 ± 2.9	0.2	1.0

### 2.2 Murine models

We studied three mouse models with targeted disruption of GCK. All mice were maintained on a 12 h dark cycle with *ad libitum* access to food and water except where indicated. All animal studies complied with ARRIVE guidelines and were performed in accordance with the U.K. Animals (Scientific Procedures) Act, 1986 (Amended 2012) and associated guidelines, EU Directive 2010/63/EU for animal experiments following ethical review by the University of Cambridge Animal Welfare and Ethical Review Body (AWERB).

#### 2.2.1 *Gck*-mutant mice

Mice with an isoleucine to phenylalanine mutation (I366F) created by ethyl-nitrosourea mutagenesis were backcrossed onto a BALBc/C3H background (Charles River, UK). Homozygote (HOM), heterozygote (HET) and wild type (WT) littermates were studied [22].

#### 2.2.2 β-cell ablated *Gck*-mutant mice

To determine whether observations from *Gck* mutant mice (and GCK-MODY humans) were related to altered GCK activity in pancreatic β-cells or other sites such as brain, we examined responses to hypoglycemia in I366F mice with β-cells ablated by streptozotocin (STZ). Starting at age 3 weeks, female I366F mice received a single ip dose of 200 mg/kg of STZ and were monitored for the development of hyperglycemia. We aimed to create a model of insulin-dependent diabetes while avoiding uncontrolled hyperglycemia in animals and matching glycemic exposure prior to studying. To achieve this, where diabetes developed, mice were treated with once daily insulin levemir (Detemir, NovoNordisk, Crawley, UK) with insulin doses adjusted in individual animals according to tail-vein sampling aiming for glucose values of approximately 15 mM. After 5 weeks of therapy, animals underwent surgery and clamp studies as described below.

#### 2.2.3 Conditional brain *Gck* knock out mice

Conditional brain *Gck* knock out (brain GCK^KO^) mice were created by crossing mice expressing Cre recombinase under the nestin promoter and CNS-specific enhancer (Nes^Cre^) with *Gck*-floxed (GK^lox^) mice (North Carolina MMRRC repository) on C57BL/6 background [23], [24].

### 2.3 Hypoglycemic Clamp Studies in Human Participants

Participants were admitted to the investigation unit at The Royal Devon and Exeter Hospital on the morning of the study after a 10 h overnight fast, having omitted any regular medication for at least 12 h prior to the test. A retrograde catheter was placed in a peripheral vein of the non-dominant hand and maintained in a heated sheepskin hand warmer to arterialize venous blood. A second catheter, for later infusion of insulin and glucose, was placed in the antecubital fossa of the same arm. At least 30 min after placing the catheters, five arterialized blood samples were drawn at 10-minute intervals for baseline measurement of glucose and counter-regulatory hormones.

Studies were performed as previously described [25]. In brief, a primed-continuous infusion of regular human insulin in 0.9% saline containing 4% autologous blood was infused at a rate of 1.5 mU kg^−1^min^−1^ for the duration of the study. Whole blood glucose was measured at the bedside every 5 min and controlled by adjustment of a simultaneous intravenous infusion of 20% glucose.

Blood glucose was reduced to 5 mmolL^−1^ from the fasting level and stabilized over the first 40 min of the clamp. It was then reduced stepwise to 4.4, 3.8, 3.4, 2.8, and 2.4 mmolL^−1^ before being restored to 5 mmolL^−1^. Each step lasted for 40 min except the nadir, which lasted for 20 min. Arterialized blood was collected for counter-regulatory hormone analysis at the midpoint, third quartile and end of each step. Symptoms were assessed using a linear analogue scale on a standard questionnaire at the midpoint and end of each glucose step.

### 2.4 Hypoglycemic clamp studies in I366F mice

For clamps, six-week old male I366F mice underwent hyperinsulinemic hypoglycemic clamp studies as previously described (but here without donor blood infusion) [26]. In summary, 1 week after surgical placement of arterial and venous catheters, mice were studied conscious and free-moving after a 6 h fast including acclimatisation to study cages. Blood sampling was performed remotely without handling mice through long lines attached to indwelling arterial catheters. Mice received a 100 mU/kg bolus of Humulin S (Eli Lilly, Basingstoke, UK) followed by a continuous infusion of 20 mU/kg/min. Because of the expected differing baseline glucose values between genotypes, plasma glucose was adjusted in all 3 groups to 5 mM during the initial 45 min and then lowered over the following 45 min to a nadir of 3 mM. At the end of studies, a sample was drawn for assay of plasma insulin (also measured at baseline), glucagon and epinephrine. To examine the potential effects of study conditions on counter-regulatory hormones, a further control group of HET mice underwent surgical catheterisation but had “dummy clamps” with blood sampling but no infusions on study days (designated SAMP).

### 2.5 Hypoglycemic clamp studies in β-cell ablated I366F mice

After 5 weeks of insulin therapy, animals underwent surgery and clamp studies as above except using single jugular venous catheters to reduce surgical time and optimise post-operative recovery in potentially fragile insulin-dependent diabetic animals. Hypoglycemic clamps were therefore performed for this study on conscious animals using a semi-restraint technique with tail vein sampling as previously described [26]. At the end of studies, pancreata were removed for assay of insulin content and compared with non-STZ controls sacrificed *ad libitum*.

### 2.6 Hypoglycemic clamp studies in brain GCK knock out mice

Three groups of male mice (brain GCK^KO^, Nes^Cre^ and GCK^lox^ controls) were studied aged 6–8 weeks. As above, overnight-fasted semi-restrained venous-catheterised mice underwent hyperinsulinemic hypoglycemic clamp studies. In these studies, we used a lower insulin dose of 10mU/kg/min, anticipating greater whole body insulin sensitivity compared with I366F mice which have altered hepatic GCK [23]. Plasma glucose was gradually reduced to approximately 3 mM and maintained at this level for 60 min. At the end of the studies, samples were collected for assay of plasma insulin, glucagon, and epinephrine.

### 2.7 Blood assays

In rodent studies, plasma glucose was measured using an Analox GM-9 analyser (Analox Instruments, London UK). Insulin and glucagon were measured by RIA (Linco) and epinephrine by ELISA (IBL Hamburg, Germany), or HPLC as previously described [27]. In human clamps, we measured whole blood glucose at bedside using a Yellow Springs glucose analyzer (Yellow Springs Instruments, Yellow Springs, Ohio); catecholamines by high performance liquid chromatography with electrochemical detection [28]; cortisol by radioimmunoassay [29]; glucagon (Diagnostic Products, Los Angeles USA), C-peptide (Linco, St Louis Mo USA) and pancreatic polypeptide using commercial radioimmunoassay kits, and growth hormone using a commercial immunoradiometric assay (Netria, London).

### 2.8 Glucose phosphorylation assay

Animals were sacrificed at the beginning of light cycle. Tissue was homogenized in lysis buffer (50 mM HEPES, 150 mM KCl, 1 mM dithiothreitol (DTT), 4 protease inhibitor cocktail tablets (Roche), pH 7.4) on ice. Homogenates were centrifuged twice (14,000 rpm for 15 min at 4 °C) and supernatant stored at −80 °C until required. Protein concentration was estimated against BSA standards using Bradford protocol [30].

Glucose phosphorylation was determined by coupling the glucose phosphorylation activity of GCK to the oxidation of glucose 6-phosphate by glucose 6-phosphate dehydrogenase with concomitant reduction of NAD^+^ to NADH detected at 340 nm. The reaction was started by adding 400 μg (peripheral tissues) or 20 μg (brain) extract to reaction cocktail (100 mM HEPES (pH 7.4), 5 mM ATP, 6 mM MgCl2, 0.05% BSA, 150 mM KCl, 1 mM DTT, 1 mM NAD^+^, 5 U/ml G6PD) supplemented with 0.5 mM and 6 mM (peripheral) or 2.5 mM (brain) glucose, to a final volume of 100 μl. Absorbance was measured at 340 nm over 60 min on a Fusion Universal Microplate Analyser at 37 °C. Glucose phosphorylating activity of GCK was then calculated by deducting the amount of glucose phosphorylation at 0.5 mM glucose (attributed to low Km hexokinase activity).

### 2.9 GCK mRNA expression in mice

Tissues were homogenised in RNA Stat-60 (Amsbio, UK) and RNA was extracted using chloroform, precipitated with isopropanol and re-suspended in RNase-free water. cDNA was synthesized from 500 ng RNA using the final reaction mix of 50 ng random hexamer primers, 1.25 mM dNTPs, 200U M-MLV reverse transcriptase (Promega, UK), 1× RT buffer (Promega) and 2.5 mM MgCl_2_ in a final volume of 20 μl. Primer sequences are detailed in Supplementary Table 1. All samples were analysed in duplicate and average cycling threshold (Ct) units were obtained as the average of the results. 18s was used as an internal control, and the other housekeeping genes were analyzed using the Bestkeeper algorithm, which was used to normalize data of the GK gene.

### 2.10 Data analysis

For human studies, we calculated peak hormone levels as the highest value achieved for each hormone. Because of the high degree of individual variability, we corrected these levels for baseline and expressed them as incremental rise over baseline for each individual. We calculated area under the curve (AUC) for each hormone between 40 and 220 min (the time of matched hypoglycemia during the clamp) as an increment above the hormone value at 40 min. We determined glucose thresholds for hormone responses as the glucose level at which there was a sustained increase of ≥3 standard deviations over the mean of five baseline measurements for a specific hormone as previously described [21]. We defined thresholds for the development of autonomic, neuroglycopenic and total (sum of autonomic and neuroglycopenic) symptoms as the plasma glucose levels at which symptom scores increased by ≥ 2 points over baseline on two consecutive assessments [31]. When no threshold was defined for an individual, we used the glucose nadir achieved during the clamp for that subject's contribution to group statistical analyses. To allow for the confounding effect of prevalent hyperglycemia on hypoglycemic responses, the main comparison was with the glycemia-matched patients with type 2 diabetes. We compared results between groups using the Mann–Whitney U test.

As described above, each of our three murine models (I366F, β-cell ablated I366F and brain GCK^KO^) were studied on different genetic backgrounds or gender, which may impact on brain transcriptome and physiological responses to hypoglycaemia [32], [33]. Statistical analyses were therefore performed within each of these three studies (comparing littermate controls studied in parallel and contemporaneously) with no statistical comparison between murine studies. Rodent data were analyzed and plotted using GraphPad Prism version 4 (GraphPad Software Inc, San Diego, California, USA). For statistical analysis, data were analyzed by either; (i) one-way ANOVA using post-hoc Tukey's to compare all pairs of columns, (ii) one-way ANOVA using post-hoc Dunnett's to compare all columns to control, (iii) two-way ANOVA with post-hoc Bonferroni test or (iv) student's t-test as appropriate. Significance level was P < 0.05 throughout. Data are expressed as means ± SEM unless otherwise stated.

## 3 Results

### 3.1 GCK-MODY humans have increased counter-regulatory responses to hypoglycemia

**Figure 1:**
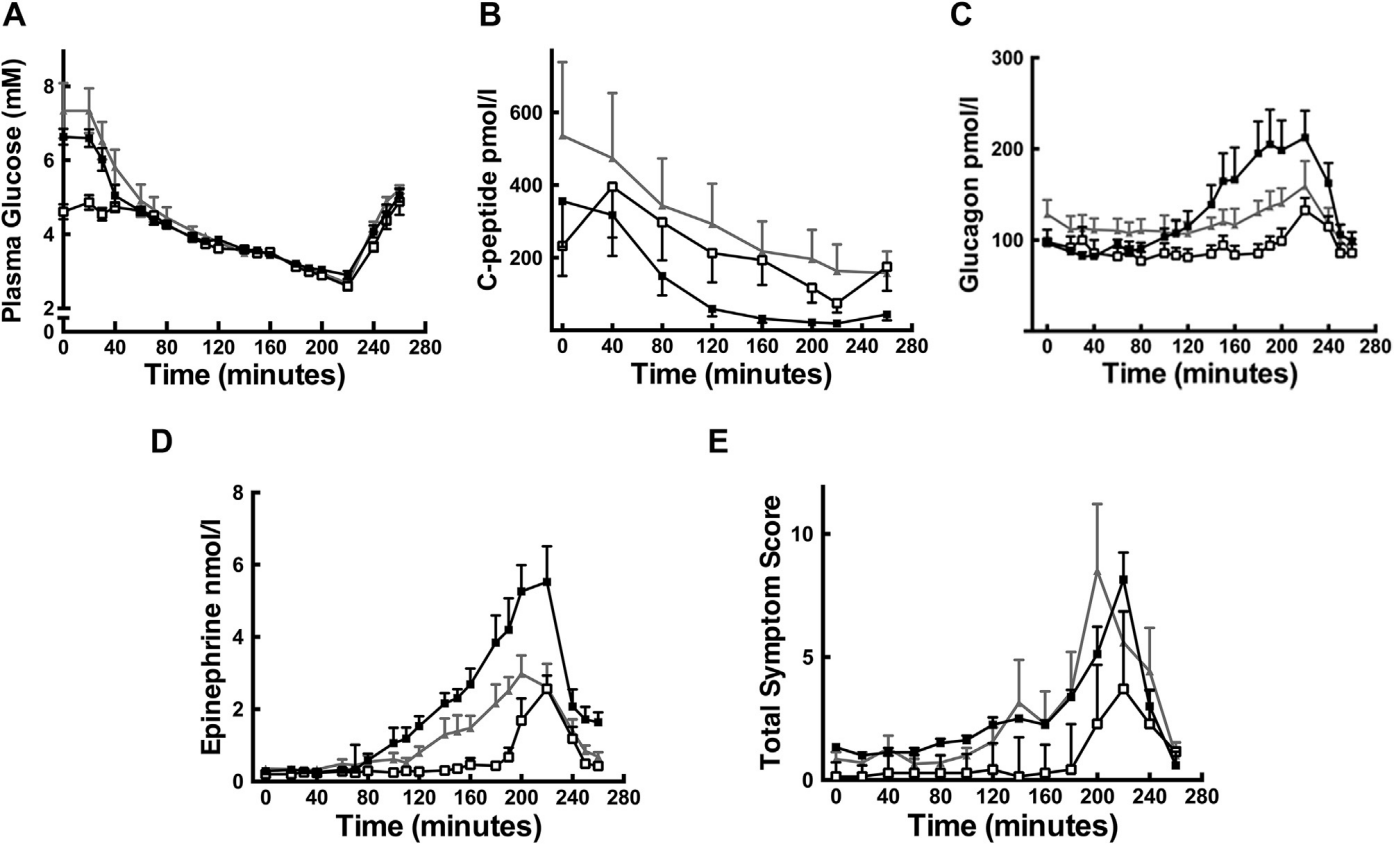
**Hypoglycemic Clamp Studies in Human Participants:** A) Whole blood glucose; B) c-peptide; C) glucagon; D) epinephrine and E) symptoms scores during the hyperinsulinemic, hypoglycemic clamp studies. Key: (GCK MODY) black squares, type 2 diabetes (type 2) grey triangles and non-diabetic controls [18] open squares, shown as mean ± SE.

Mean blood glucose profiles of the three groups obtained during the clamp are shown in Figure 1A. Importantly, there was no significant difference in fasting glucose between GCK-MODY (6.6 ± 0.6  mmol L^−1^) and T2D groups (6.7 ± 0.9  mmol L^−1^, p = 0.8) nor at any step during the clamp. As expected, baseline fasting blood glucose values were lower in healthy controls (4.6 ± 0.6 mmolL^−1^, p = 0.002 vs GCK-MODY; p = 0.003 vs T2D). From 40 min onwards in clamp studies, there were no significant differences in blood glucose values between the three groups. Exogenous glucose infusion requirements during clamp studies were similar in the GCK-MODY (394 ± 249 mmol) and T2D (232 ± 212 mmol; p = 0.2) groups with both groups requiring significantly less glucose during the course of the clamp compared to healthy controls (903 ± 449 mmol, p = 0.03 vs GCK-MODY; p = 0.007 vs T2D).

The GCK-MODY group had an earlier onset (higher glucose at threshold of glucagon release; p = 0.007) and greater peak glucagon response (p = 0.03) than the T2D group (Table 2a and Figure 1C). Compared with healthy controls, the GCK-MODY group also had an earlier onset (p = 0.002), greater peak incremental glucagon response (p = 0.046) and greater AUC (p = 0.01). The GCK-MODY group also had a greater peak epinephrine rise (p = 0.03) and AUC for epinephrine (p = 0.005) compared with the T2D group (Tables 2a and b and Figure 1D). Compared with the healthy controls, the GCK-MODY group had earlier onset (p = 0.009) and greater incremental peak (p = 0.03) and AUC (p = 0.001) for epinephrine responses (Table 2a and b & Figure 1D). There was no difference in glucose threshold (p = 0.07), AUC or peak epinephrine response between the between the T2D groups and healthy controls (Tables 2a and b and Figure 1D).

**Table 2a:** Peak incremental counter-regulatory hormone rise above baseline ±SD.

	GCK-MODY	Type 2 diabetes	p-value GCK-MODY vs Type 2 diabetes	Healthy controls	p-value GCK-MODY vs Healthy controls	p-value Type 2 diabetes vs Healthy controls
Glucagon/pg ml^−1^	127 ± 88	42 ± 30	0.03	43 ± 40	0.046	1.0
Epinephrine/nmol L^−1^	5.5 ± 2.5	2.8 ± 1.4	0.03	2.9 ± 1.9	0.03	0.8
Norepinephrine/nmol L^−1^	2.4 ± 1.9	1.7 ± 1.1	0.5	1.6 ± 0.7	0.5	0.7
Cortisol/nmol L^−1^	540 ± 249	458 ± 197	0.6	312 ± 142	0.046	0.12
Growth Hormone/mu L^−1^	42 ± 50	22 ± 13	0.4	44 ± 21	0.1	0.03
Pancreatic polypeptide/pmol L^−1^	192 ± 120	343 ± 275	0.4	165 ± 208	0.5	0.6

**Table 2b:** Incremental Area Under the curve (AUC) for counter-regulatory hormone release (from 40 to 220 min) ± SD.

	GCK-MODY	Type 2 diabetes	p-value GCK-MODY vs Type 2 diabetes	Healthy controls	p-value GCK-MODY vs Healthy controls	p-value Type 2 diabetes vs Healthy controls
Glucagon/pg ml^−1^	10,070 ± 8929	984 ± 5412	0.01	416 ± 4148	0.01	0.8
Epinephrine/nmol L^−1^	354 ± 176	136 ± 51	0.005	69 ± 65	0.001	0.09
Norepinephrine/nmol L^−1^	117 ± 166	62 ± 35	0.8	26 ± 40	0.07	0.09
Cortisol/nmol L^−1^	36,269 ± 34,048	25,858 ± 35,477	0.6	1973 ± 17,044	0.02	0.09
Growth Hormone/mu L^−1^	2550 ± 2159	1291 ± 799	0.2	445 ± 1345	0.04	0.2
Pancreatic polypeptide/pmol L^−1^	9632 ± 5992	12,000 ± 8880	0.7	1261 ± 1294	0.001	0.003

There was no difference between the GCK-MODY and T2D groups for other measured counter-regulatory hormones (Tables 2b and c). C-peptide suppression was greater in patients with GCK-MODY compared to T2D and healthy controls at each time point from 120 min (Figure 1B) (GCK vs T2D, p = 0.04; GCK vs healthy, p = 0.02) to 260 min (GCK vs T2D, p = 0.007; GCK vs healthy, p = 0.006). There were no differences in the glucose threshold of total, autonomic or neuroglycopenic symptoms of hypoglycemia between the GCK-MODY and T2D groups (Table 2c and Figure 1E). The GCK-MODY had earlier onset of total (p = 0.002) autonomic (p = 0.002) and neuroglycopenic (p = 0.02) symptoms compared with healthy controls (Table 2c).

**Table 2c:** Glucose thresholds for counter-regulatory hormone release and total symptoms ± SD.

	GCK-MODY (mmol L^−1^)	Type 2 diabetes (mmol L^−1^)	p-value GCK-MODY vs Type 2 diabetes	Healthy controls (mmol L^−1^)	p-value GCK-MODY vs Healthy controls	p-value Type 2 diabetes vs Healthy controls
Glucagon	3.6 ± 0.5	2.9 ± 0.3	0.007	2.6 ± 0.3	0.002	0.12
Epinephrine	4.0 ± 0.6	3.7 ± 0.5	0.4	3.2 ± 0.9	0.009	0.07
Norepinephrine	3.2 ± 0.5	3.5 ± 0.5	0.4	2.7 ± 0.4	0.03	0.01
Cortisol	3.7 ± 0.9	3.6 ± 1.2	0.6	2.7 ± 0.5	0.02	0.09
Growth Hormone	3.6 ± 0.5	3.6 ± 0.3	0.6	2.6 ± 1.2	0.02	0.01
Pancreatic polypeptide	3.5 ± 0.5	3.5 ± 0.3	1.0	2.8 ± 0.5	0.01	0.005
Total symptoms	3.6 ± 0.7	3.5 ± 0.9	0.8	2.6 ± 0.3	0.002	0.02
Autonomic symptoms	3.5 ± 0.4	3.4 ± 0.9	0.4	2.6 ± 0.3	0.002	0.02
Neuroglycopenic symptoms	3.0 ± 0.4	2.9 ± 0.4	0.4	2.6 ± 0.3	0.02	0.16

In summary, data from GCK MODY humans were consistent with our hypothesis that GCK-mediated sensing of hypoglycemia contributes to the integrated glucagon and epinephrine counter-regulatory response to a falling blood glucose. Given the whole body GCK mutations seen in GCK MODY patients, we then aimed to examine further the contributions from different glucose sensing sites by using murine models.

### 3.2 I366F GCK mutant mice show amplified glucagon response to hypoglycemia

**Figure 2:**
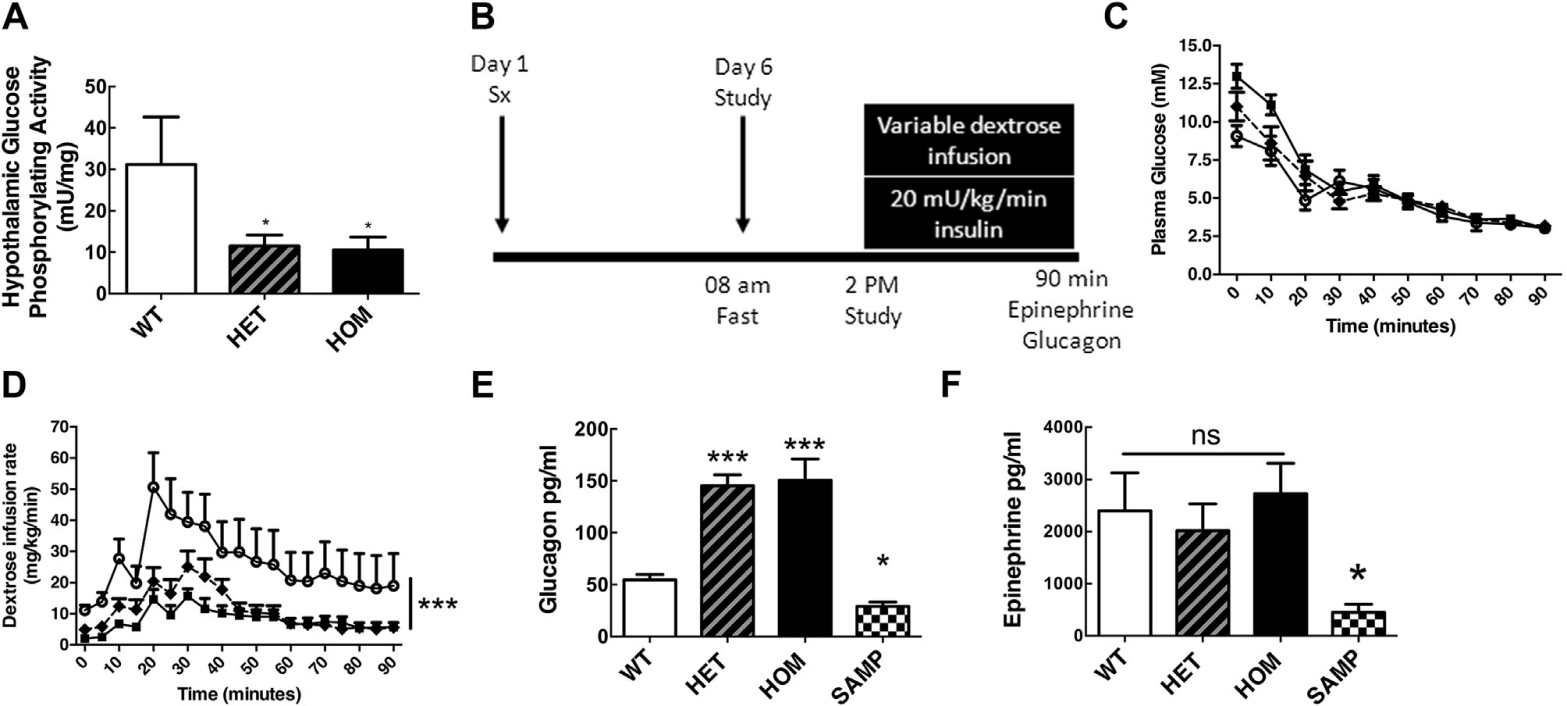
**Hypoglycemic clamp studies in I366F mice.** (A) Effect of GK gene mutation on hypothalamic glucose phosphorylating activity of I366F mice. (B) Schematic of study design from surgery on day 1 through to hypoglycemic clamp on day 6. (C) Plasma glucose and (D) dextrose infusion rates during hypoglycemic clamp studies. (E) Plasma glucagon and (F) epinephrine responses at the end of hypoglycemic clamp studies. White circles = WT; black diamonds/dashed line = HET; black squares = HOM. ‘SAMP’ represents dummy clamp mice with sampling only. All data are from ∼8-week-old male mice presented as mean ± SEM, n = 6–10 in each group, *p < 0.05, **p < 0.01, ***p < 0.001. See also Supplementary Figure 1.

**Supplementary Figure 1:**
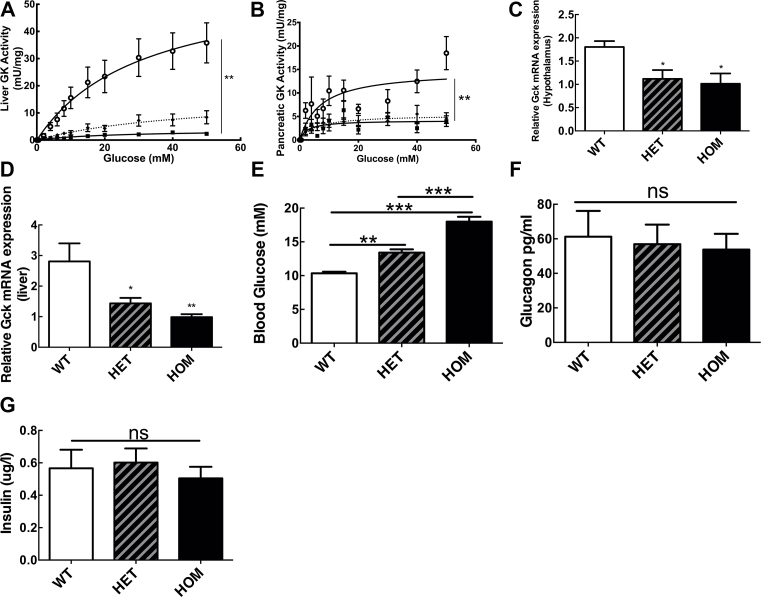


We first examined I366F mice with a point mutation in GCK. As expected in this whole body GCK-mutant mouse model, we found reduced glucose phosphorylating activity in liver and pancreas of both heterozygous (HET) and homozygous (HOM) I366F mice compared to wild type (WT) littermates (Supplementary Figures 1a and b). Glucose phosphorylating activity measured *ex vivo* at a physiological brain-glucose level (2.5 mM) was lower in hypothalamus of both HET and HOM I366F mice compared to WT (Figure 2A). Quantitative PCR also showed reduced GCK mRNA levels in hypothalamus and liver of both HET and HOM mice compared to WT (Supplementary Figures. 1c and d). Analogous with human GCK-MODY, *ad libitum* blood glucose was elevated in mice with mutant GCK (both HET and HOM in mice) with no compensatory changes in insulin or glucagon (Supplementary Figures 1e–g).

During clamp studies (Figure 2B), plasma glucose values were well matched in all genotypes from 45 min onwards (Figure 2C). HOM and HET mice required significantly less dextrose than WT mice, likely due to hepatic insulin resistance/reduced hepatic glucose uptake secondary to mutant GCK (Figure 2D). Consistent with our hypothesis, I366F HOM mice showed increased glucagon responses to acute hypoglycemia (Figure 2E). This increase was specific for hypoglycemia, as levels were not raised in non-hypoglycemic mice (SAMP). In these studies, there was no effect of genotype on epinephrine responses (Figure 2F).

### 3.3 Amplified hypoglycemia counter-regulation in I366F GCK mutant mice is not due to abnormal β-cell GCK

**Figure 3:**
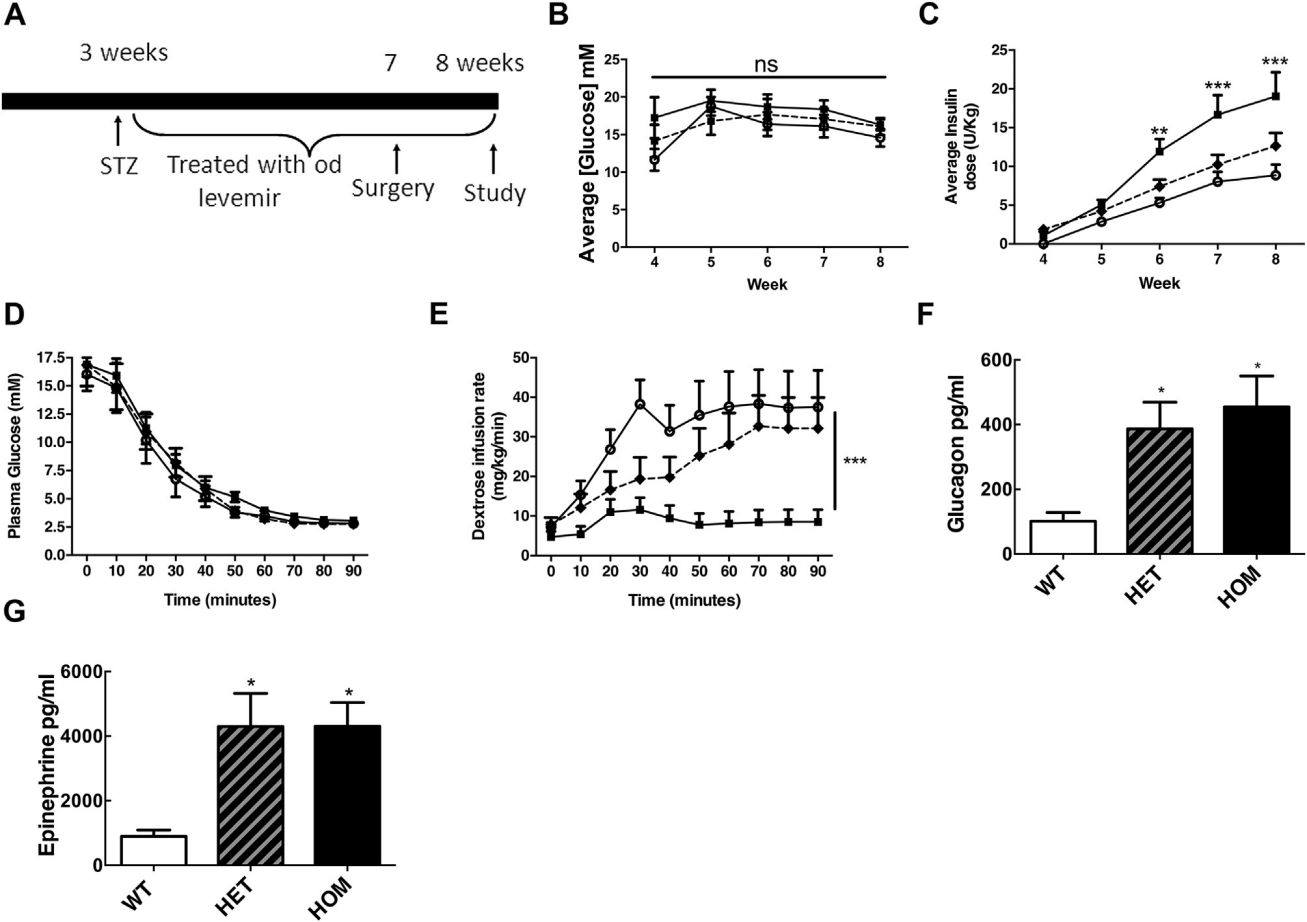
**Hypoglycemic clamp studies in beta-cell ablated I366F Mice.** (A) Schematic diagram of study design with 200 mg/kg of streptozotocin given I.P. at weaning. (B) Based on blood glucose sampling from the tail vein, (C) O.D. insulin detemir (levemir) doses were titrated. (D) Plasma glucose profile and (E) dextrose infusion rates during hypoglycemic clamp studies. (F) Plasma glucagon and (G) epinephrine responses to clamped hypoglycemia. White circles = WT, black diamonds/dashed line = HET, black squares = HOM. Clamp data are from ∼8-week-old male mice, presented as mean ± SEM, n = 9–11 in each group, *p < 0.05, **p < 0.01, ***p < 0.001. See also Supplementary Figure 2.

GCK-MODY humans and I366F mice have a whole body GCK mutation and it is possible that abnormal β-cell GCK and reduced intra-islet paracrine (insulin) signalling contribute to increased glucagon responses. To address this, we examined responses to hypoglycemia in I366F mice that had undergone β-cell ablation with high dose STZ. Importantly, we subsequently treated animals with insulin to match carefully glycemia in all genotypes. As anticipated, STZ therapy resulted in almost undetectable pancreatic insulin content (Supplementary Figure 2). By careful titration of daily insulin dosing, we were able to match blood glucose levels after STZ therapy for 4 weeks prior to clamp studies in all genotypes (Figure 3A,B). Insulin doses were individualized and HOM mice needed more insulin than other genotypes to achieve similar glycemia (Figures 3C), likely explained at least in part by altered hepatic substrate flux as described previously in hepatic *Gck* KO mice [23].

During clamp studies in STZ-treated I366F mice, starting plasma glucose values similar in all groups and well matched during studies (Figure 2D). Plasma insulin values at baseline were similar (2.6 ± 0.9, 2.5 ± 0.8, 2.0 ± 0.2 ug/l; WT, HET, HOM) and rose during clamp studies with no differences between groups (peak values 17 ± 4, 27 ± 10, 22 ± 3 ug/l; WT, HET, HOM respectively). HET and HOM mice required less dextrose during clamp studies to achieve similar glucose profiles (Figure 3E). As anticipated, HET and HOM mice demonstrated significantly greater glucagon and epinephrine responses to hypoglycemia compared with WT (Figure 3F,G). In summary, these data show that the increased glucagon counter-regulatory response to hypoglycemia in mutant I366F mice is not simply due to altered GCK activity (and insulin release/glycemia) in pancreatic β-cells.

### 3.4 Conditional brain GCK^KO^ mice have an amplified counter-regulatory response to hypoglycemia

**Figure 4:**
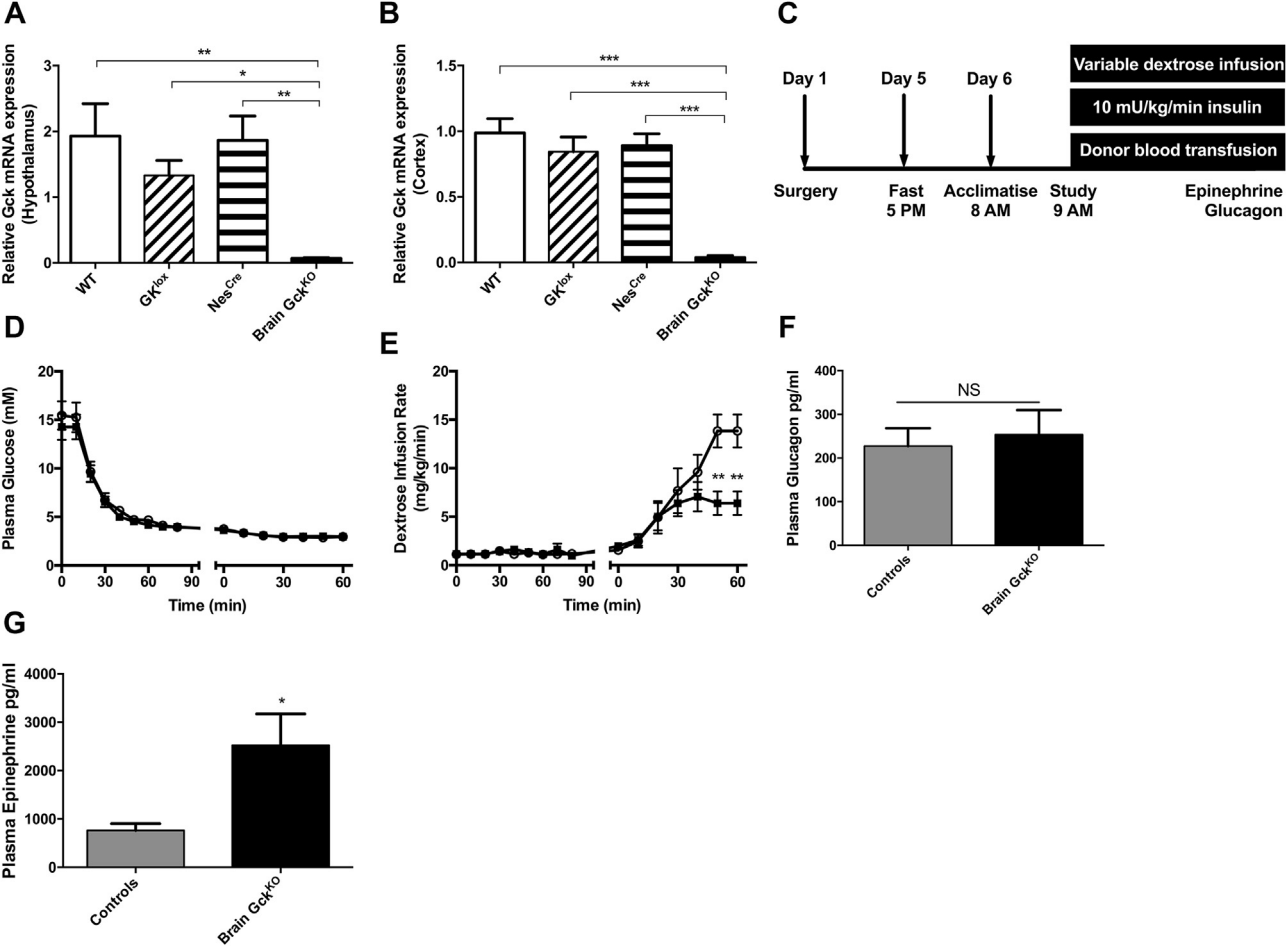
**Hypoglycemic clamp studies in conditional Brain GCK**^**KO**^**mice.** (A–B) GCK mRNA expression level in hypothalamus and cortex of Brain GCK^KO^ mice and control littermates. (C) Schematic diagram of hypoglycemic clamp design in conditional Brain GCK^KO^ mice. (D) Plasma glucose profile and (E) Dextrose infusion rates during hypoglycemic clamp studies. (F) Plasma glucagon and (G) epinephrine responses to clamped hypoglycemia. Black squares, Brain GCK^KO^; open circle, controls. Control represents pooled group of GCK^lox^ and Nes^Cre^ mice. Data presented as mean ± SEM in 6–8-week old mice, n = 5 in each group for qRT-PCR data and 13 to 14 in each group for clamp studies, *p < 0.05, **p < 0.01, ***p < 0.001.

To examine further the distinct role for brain GCK in hypoglycemia counter-regulation, we studied a conditional brain GCK^KO^ mouse model. First, we confirmed the absence of brain *Gck* mRNA in brain GCK^KO^ mice (Figure 4A,B). During hypoglycemic clamp studies in brain GCK^KO^ mice, there were no differences between GCK^lox^ and Nes^Cre^ control groups for any parameters examined; thus, clamp data are shown as brain GCK^KO^ versus combined controls (pooled GCK^lox^ and Nes^Cre^). Plasma glucose levels were well matched in brain GCK^KO^ and control groups throughout the clamp studies (Figure 4D). Baseline counter-regulatory hormone levels were similar (glucagon 30 ± 3 *vs* 25 ± 2 pg/ml; epinephrine 1153 ± 269 *vs* 1370 ± 303 pg/ml; controls *vs* brain GCK^KO^). Plasma insulin levels at baseline were also comparable (0.1 ± 0.1 *vs* 0.1 ± 0 ug/l; controls *vs* GCK^KO^) and rose during clamp studies to a peak value with no difference between groups (2.0 ± 0.3 *vs* 1.4 ± 1.4 ug/l; controls *vs* brain GCK^KO^). Brain GCK^KO^ mice required significantly less dextrose to maintain hypoglycemia during the late phase of clamp studies (Figure 4E), suggesting amplified counter-regulatory responses to hypoglycemia. In keeping with this, brain GCK^KO^ mice showed a higher epinephrine response to acute hypoglycemia compared to controls (Figure 4G). No differences in glucagon response were observed (Figure 4F).

## 4 Discussion

Our data reveal that a GCK-dependent glucose-sensing mechanism operates during hypoglycemia in both humans and mice. This mechanism facilitates three key protective responses to a falling glucose, being involved in (i) the hypoglycemia-associated reduction in insulin secretion and release of the counter-regulatory hormones (ii) glucagon and (iii) epinephrine.

The ability to switch off endogenous insulin release during falling blood glucose is an important and sometimes under-appreciated defence against hypoglycemia. Consistent with an important role for GCK in beta cell glucose-sensing, humans with GCK-MODY showed greater suppression of C-peptide beginning at low normal blood glucose concentrations (from 3.8 mmolL^−1^) compared with T2D and healthy controls. While not unexpected that insulin secretion is influenced by the presence of a *GCK* mutation, to our knowledge, this is the first time that a different effect on endogenous insulin secretion between GCK-MODY and T2D has been demonstrated *in vivo* during acute hypoglycemia.

Supporting a role for GCK in mediating glucagon release, we observed increased glucagon responses in GCK-MODY and I366F mice with whole body reduced GCK activity. A previous study of GCK-MODY also reported increased glucagon (but not epinephrine) responses during experimental hypoglycemia, although hypoglycemic challenges were not matched between groups [34]. Glucagon release from α-cells during hypoglycemia may be triggered both by direct α-cell sensing of glucose, local paracrine signalling from within islets and distal factors such as autonomic pancreatic innervation and circulating factors such as catecholamines [8]. This means that intra-islet GCK found in α- and β-cells and GCK at distal sensing sites such as brain could all potentially contribute to counter-regulatory glucagon responses. Here, we found that increased glucagon responses to hypoglycemia persisted in I366F mice following β-cell ablation, suggesting that local paracrine signalling from pancreatic β-cells was not necessary to mediate effects of mutant GCK. We saw no differences in glucagon responses in our brain GCK^KO^ model suggesting that brain GCK was not predominantly involved in glucagon responses to hypoglycemia. Taken together, these data are consistent with direct GCK-mediated sensing of hypoglycemia by α-cells being an important part of the counter-regulatory glucagon response. In keeping with this, a recent paper described mice with α-cell GCK knockout displaying increased glucagon levels, although responses to hypoglycemia were not reported [35]. Our findings do not preclude other non-GCK brain glucose sensing, for example using GLUT-2, contributing to the central glucagon response [36], [37].

Epinephrine responses to hypoglycemia are probably triggered centrally, perhaps with input from peripheral hypoglycemia sensing [3], [4]. We observed increased epinephrine responses in GCK-MODY and I366F diabetic mice. Consistent with a role for brain GCK in detecting a low blood glucose, we also saw increased epinephrine responses to hypoglycemia in brain GCK^KO^ mice. It is unclear why there were no observed differences in epinephrine responses to hypoglycemia in I366F mice given the findings in the other 3 models studied. The clamp protocol in I366F mice was different from that used for I366F diabetic and brain GCK^KO^ mice. However, we would anticipate that the double catheter technique with mice being free moving and unhandled would carry less potential for the confounding effects of stress from study conditions. In these murine studies, we were limited by sampling volumes to a single measure of counter-regulation at the end of studies and it is possible that a more complete time series might have unmasked differences.

Brain GCK expression is restricted to key brain glucose-sensing areas including areas in the basomedial hypothalamus such as the ARC and VMN [16], [17], [18], [19]. A recent study examined electromagnetic inhibition of VMN GCK-neurons, finding blunted hyperglycemic response to 2-deoxyglucose induced glucopenia (although individual counter-regulatory hormone responses were not reported) [38]. A previous study used short hairpin RNA-knockdown of VMN GCK in rats, reporting increased epinephrine responses to non-clamped (insulin-bolus induced) hypoglycemia [39]. It is possible that VMN neurons involved in GCK-mediated counter-regulation will be non-SF1 cells given that genetic inactivation of GCK specifically within VMN SF1 cells does not increase epinephrine or glucagon release in response to hypoglycemia [40].

We did not see significantly different responses between GCK-MODY and T2D groups for the other measured counter-regulatory hormones in human studies. This suggests either that GCK is less important in these responses and/or that adaptation occurs. Transcriptional profiling of hypothalamic GCK cells in a model revealed a population of hypoglycemia-activated growth hormone releasing hormone cells, suggesting a role for brain GCK in generating growth hormone responses to hypoglycemia [20]. It is unclear if this reflects species differences. Given the volumes required for sampling, it was not possible to measure additional hormones in our mice models.

Two studies in humans examined brain GCK and hypoglycemia indirectly, using fructose infusion and hypothesizing that this acted via increased brain fructose-6 phosphate to activate GCK regulatory protein (GKRP), leading in turn to inhibition of brain GCK. Systemic fructose infusion amplified glucagon and epinephrine responses to hypoglycemia in healthy subjects and increased epinephrine responses in patients with type 1 diabetes [41], [42]. However, it is unclear whether brain GCK inhibition is indeed the mechanism of action of fructose which may have other actions such as hypothalamic AMPK activation [43]. The strengths of our experimental approach are that we studied defined molecular perturbations in GCK and used insulin clamp techniques across all models to create carefully controlled and matched hypoglycemic challenges (as opposed to insulin bolus-induced hypoglycemia or glucoprivation).

Of note, each of our three murine models (I366F, β-cell ablated I366F and brain GCK^KO^) were studied on different genetic backgrounds and/or gender. Previous data have shown quantitative differences between strains in the magnitude and threshold of counter-regulatory responses to hypoglycemia [32]. We used female mice in our streptozotocin studies to maximize efficient use of the I366F breeding colony. Gender differences in counter-regulation have been reported in human and murine studies [40], [44]. It is thus possible that background strain and/or gender might have altered our findings.

A number of GCK activators for treating T2D have been developed and tested but not progressed successfully from clinical trials into therapy [45]. Our observations suggest that increased risk of hypoglycemia might be possible, particularly if drugs penetrate into brain. A further possibility is that GCK inhibition could offer a therapeutic approach to increase hypoglycemia counter-regulatory defences in diabetes.

Our findings also provide a physiological explanation for observations seen in clinical practice when treating GCK-MODY diabetes, where there is a reduced response to exogenous insulin including during pregnancy [46], [47]. We found that insulin suppression and glucagon release occur at a higher glucose levels in GCK-MODY than T2D and that there is an exaggerated epinephrine response to glucose lowering in GCK-MODY. This supports the clinical observations that large doses of insulin may be needed to achieve pregnancy glucose targets in women with GCK-MODY and those who reach pregnancy glucose targets frequently report autonomic symptoms of hypoglycemia [personal communications ATH & MH Shepherd].

## 5 Conclusions

Our data identify a GCK-dependent glucose-sensing mechanism during hypoglycemia that boosts responses to falling glucose; augmenting hypoglycemia-associated reduction in insulin secretion and the release of the counter-regulatory hormones glucagon and epinephrine. Moreover, we reveal a specific function of GCK in defined sub-regions that regulate these three effects. Our data suggest that β-cell GCK is responsible for insulin suppression as blood glucose levels fall, whereas GCK outside the β-cell (likely in pancreatic α-cells) is necessary for a normal glucagon response and GCK within brain glucose-sensing areas contributes to epinephrine responses to hypoglycemia. These findings are relevant to the treatment of the growing global diabetes epidemic.

## Figures and Tables

**Supplementary Figure 2:**