# Supplementary material for: Molecular reductions in glucokinase activity increase counter-regulatory responses to hypoglycemia in mice and humans with diabetes
Source: Mol Metab. 2018 Aug 13;17:17–27. doi: 10.1016/j.molmet.2018.08.001 (PMC6197723; doi:10.1016/j.molmet.2018.08.001)
Supplement: Multimedia component 1 [file mmc1.docx]

**Supplementary Information**

**Supplementary Figure 1: *Ex vivo* GK assays and *ad libitum* blood sampling in I366F mice.**

(A) Reduced glucose phosphorylating activity in liver and (B) Pancreas of HET and HOM I366F mice.

(C) Reduced GK mRNA expression in hypothalamus and (D) liver of HET and HOM I366F mice.

*Ad libitum* light cycle sampling revealed hyperglycemia in HET and HOM I366F mice compared to WT littermates (E) but no difference in plasma glucagon (F) and insulin (G). All data are from ~8-week-old male mice presented as mean ± SEM, n= 6-10 (A-D) and 15-20 (E-G) in each group, * p<0.05, ** P<0.01, *** P<0.001.

**Supplementary Figure 2: Pancreatic insulin content in I366F mice following streptozotocin therapy**. All data are from ~8-week-old male mice, with pancreata taken after hypoglycemic clamp study, presented as mean ± SEM, n= 6-8 in each group, *** p<0.001.

**Supplementary Table 1: Details of mutations in GCK-MODY group**.

RefSeq number NM_111162.3. * = sisters

**Supplementary Table 2: Primers used for RT-PCR**. Each PCR reaction contained cDNA (diluted from reverse transcription by 1:6 for glucokinase analysis and 1:75 for housekeeping gene analysis), SYBR green PCR master mix and 0.5μM forward and reverse primers.

**Supplementary Online Table 1:**

| **GCK Mutations in GCK-MODY group:** |
| --- |
| **c.1174C>T p.(Arg392Cys) *** |
| **c.1174C>T p.(Arg392Cys) *** |
| **c.97_117dup p.V33_K39dup** |
| **c.449T>C p.(Phe150Ser)** |
| **c.483+2_483+16del (p.?)** |
| **c.781G>A p.(Gly261Arg)** |
| **c.895G>C p.(Gly299Arg)** |
| **c.1145G>A p.(Cys382Tyr)** |

**Supplementary Online Table 2:**

Primers used for RT-PCR. Each PCR reaction contained cDNA (diluted from reverse transcription by 1:6 for glucokinase analysis and 1:75 for housekeeping gene analysis), SYBR green PCR master mix and 0.5μM forward and reverse primers.

| **Gene** | **Forward Sequence** | **Reverse Sequence** |
| --- | --- | --- |
| Glucokinase | 5-GTGAGGTCGGCATGATTGT-3 | 5-TCCACCAGCTCCACATTCT-3 |
| 18s | 5-CGGCTACCACATCCAAGGAA-3 | 5-GCTGGAATTACCGCGGCT-3 |
| 36B4 | 5-AGATGCAGCAGATCCGCAT-3 | 5-GTTCTTGCCCATCAGCACC-3 |
| Beta-2 microglobulin | 5-GGTCTTTCTCTGGTGCTTGTC-3 | 5-GTATGTTCGGCTTCCCATTC-3 |
| Beta-actin | 5-GCTCTGGCTCCTAGCACCAT-3 | 5-GCCACCGATCCACACAGAGT-3 |
